# CD147 Promotes Tumorigenesis via Exosome-Mediated Signaling in Rhabdomyosarcoma

**DOI:** 10.3390/cells11152267

**Published:** 2022-07-22

**Authors:** Assil Fahs, Nader Hussein, Hasan Zalzali, Farah Ramadan, Farah Ghamloush, Hani Tamim, Mahmoud El Homsi, Bassam Badran, Fouad Boulos, Ayman Tawil, Sandra E. Ghayad, Raya Saab

**Affiliations:** 1 Department of Biology, Faculty of Science II, Lebanese University, Fanar P.O. Box 90656, Lebanon; assil.fahs@duke.edu (A.F.); farah.ramadan@etu.univ-lyon1.fr (F.R.); 2 Laboratory of Cancer Biology and Molecular Immunology, Department of Chemistry and Biochemistry, Faculty of Science I, Lebanese University, Hadat 1003, Lebanon; nader.hussein@ul.edu.lb (N.H.); mahmoud.homsi@ul.edu.lb (M.E.H.); bassam.badran@ul.edu.lb (B.B.); 3 Department of Anatomy, Cell Biology and Physiology, Faculty of Medicine, American University of Beirut, Beirut 1107 2020, Lebanon; 4 Department of Pediatrics & Adolescent Medicine, American University of Beirut Medical Center, Beirut 1107 2020, Lebanon; hz13@aub.edu.lb (H.Z.); fg07@aub.edu.lb (F.G.); 5 Department of Internal Medicine, American University of Beirut Medical Center, Beirut 1107 2020, Lebanon; htamim@aub.edu.lb; 6 College of Medicine, Alfaisal University, Riyadh 11564, Saudi Arabia; 7 Department of Pathology and Laboratory Medicine, American University of Beirut Medical Center, Beirut 1107 2020, Lebanon; bfouad@wustl.edu (F.B.); at04@aub.edu.lb (A.T.); 8 C2VN, INSERM 1263, INRAE 1260, Aix-Marseille University, CEDEX 5, 13385 Marseille, France

**Keywords:** rhabdomyosarcoma, exosomes, CD147, tumorigenesis

## Abstract

Rhabdomyosarcoma (RMS) is an aggressive childhood soft-tissue tumor, with propensity for local invasion and distant metastasis. Exosomes are secreted vesicles that mediate paracrine signaling by delivering functional proteins and miRNA to recipient cells. The transmembrane protein CD147, also known as Basigin or EMMPRIN, is enriched in various tumor cells, as well as in tumor-derived exosomes, and has been correlated with poor prognosis in several types of cancer, but has not been previously investigated in RMS. We investigated the effects of CD147 on RMS cell biology and paracrine signaling, specifically its contribution to invasion and metastatic phenotype. CD147 downregulation diminishes RMS cell invasion and inhibits anchorage-independent growth in vitro. While treatment of normal fibroblasts with RMS-derived exosomes results in a significant increase in proliferation, migration, and invasion, these effects are reversed when using exosomes from CD147-downregulated RMS cells. In human RMS tissue, CD147 was expressed exclusively in metastatic tumors. Altogether, our results demonstrate that CD147 contributes to RMS tumor cell aggressiveness, and is involved in modulating the microenvironment through RMS-secreted exosomes. Targeted inhibition of CD147 reduces its expression levels within the isolated exosomes and reduces the capacity of these exosomes to enhance cellular invasive properties.

## 1. Introduction

Rhabdomyosarcoma (RMS) is the most common soft-tissue sarcoma in children and adolescents; it is considered rare in adults [1]. It consists of rhabdomyoblasts that fail to complete differentiation into mature skeletal muscle cells but nonetheless express myogenic transcription factors that control skeletal muscle differentiation [2]. RMS is classified, based on histology, into several subtypes. The most common are the embryonal (ERMS) and alveolar (ARMS) subtypes [3]. In pediatric and adolescent patients, ERMS constitutes around 60% of RMS and is usually associated with localized disease, favorable sites of onset and better prognosis, while ARMS is less frequent (20%) but more aggressive due to less favorable sites of primary onset and enhanced metastasis potential [1,4]. In adults, prognosis seems to be worse for both subtypes, and the prevalence of invasive and metastatic disease is higher [1]. The driver oncogene in ARMS is the product of a chromosomal translocation that results most commonly in the fusion oncoprotein PAX3-FOXO1, and less frequently PAX7-FOXO1 [4,5,6]. Additional rare fusion genes have been identified as well, such as *FOXO1-FGFR1* and *PAX3-NCO1* [7,8], among others [9]. Cases with histologically defined ARMS that lack this fusion oncoprotein are associated with clinical and molecular profiles that are indistinguishable from ERMS cases, indicating a more favorable prognosis [9,10]. This has allowed a better classification of RMS into fusion-positive (FPRMS) and fusion-negative (FNRMS) subtypes, which improves prognostication [11,12,13]. The fusion oncoprotein has been well documented to act as a transcription factor that modifies gene expression resulting in enhancement of cell survival, motility and invasion [14].

RMS cells release exosomes that can modulate the tumor microenvironment in such a way that enhances not only RMS growth but also normal fibroblasts [15]. Exosomes are extracellular nanovesicles released by normal cells, such as stem cells and immune cells, and at much higher quantities by abnormal cells, including cancer cells [16,17,18]. Exosomes carry miRNA and proteins that can modulate recipient cell function by releasing this cargo into target cells and modifying intracellular signaling pathways. In cancer, exosomes were shown to enhance evasion of the immune response, promote recipient cell motility and create a pre-metastatic niche that allows distant metastasis [18,19]. In RMS, exosomes derived from both ERMS and ARMS cell lines were found to enhance recipient fibroblast proliferation, migration and invasion [15]. Furthermore, RMS-derived exosomes were found to carry nucleic acids and proteins previously implicated in tumor growth and metastasis such as *miR-486* and integrins [20,21,22]. Proteomic profiling of RMS-derived exosomes revealed that they commonly express a set of proteins including CD147, which is enriched in exosomes of both ARMS and ERMS subtypes [20].

CD147, also known as EMMPRIN or Basigin, is a transmembrane glycoprotein and member of the immunoglobulin superfamily [23]. Its expression is upregulated in tumor cells and has been correlated with a poor prognosis in several types of cancer, including breast cancer [24,25] and melanoma [26]. CD147 is able to induce the production of matrix metalloproteinases, including MMP-1 and MMP-9, which leads to the remodeling of the extracellular matrix and enhances motility [23]. CD147 can also promote angiogenesis by increasing the protein expression levels of MMPs as well as vascular endothelial growth factor (VEGF) [27,28]. Moreover, CD147 can interact with integrins to regulate the adhesion with extracellular matrix proteins [29,30]. CD147 also participates in inflammation, nutrient and drug transporter activity and developmental processes, making it a pleotropic molecule whose expression is not limited to tumor cells [23,24,25,27,28,29,30,31]. Interestingly, evidence suggests that CD147-containing extracellular vesicles, including exosomes, can be extracted from the serum of cancer patients and may serve as a biomarker of disease [31,32,33,34]. In this study, we investigate the role of CD147 in RMS cells, and its potential contribution to the RMS microenvironment through exosome-mediated signaling.

## 2. Material and Methods

### 2.1. Cell Lines and Cell Culture

The human ERMS cell line JR1, and the ARMS cell line Rh41 were generously donated by Dr. Peter Houghton and St Jude Children’s Research Hospital. BJ (human foreskin fibroblast) cell line was purchased from ATCC (Manassas, VA, USA). The cells were cultured in Dulbecco’s modified Eagle’s medium (DMEM) AQ supplemented with 10% fetal bovine serum (FBS) and 1% penicillin (100 units/mL)—streptomycin (100 μg/mL) antibiotics (all from Sigma-Aldrich, Dorset, UK). For the ARMS cell lines, the medium was supplemented with 1% sodium pyruvate and 1% non-essential amino acids (Sigma-Aldrich, Dorset, UK). Cells were maintained under standard incubation conditions (humidified atmosphere, 95% air, 5% CO_2_, 37 °C) and passed two times per week by trypsinization using trypsin-EDTA (Sigma-Aldrich, Dorset, UK).

### 2.2. Viral Transduction

RMS cell lines were transduced with a lentiviral vector expressing *GFP* along with puromycin resistance gene and one of four different shRNA constructs directed against CD147 (*shCD147*), or scrambled control (*shScr*), all purchased from OriGene (Rockville, MD, USA). Cells were plated at a density of 1 million cells per 6 well plates, and 1 mL of virus was added with volume completed to a total of 2 mL per well by adding medium. Polybrene 8 µg /mL (Sigma-Aldrich, Dorset, UK) was added to increase the efficiency of transduction and the plate was centrifuged at 1250× *g* for 1 h followed by incubation overnight at 37 °C. The next day, the cells were trypsinized and transferred to 6 cm plates, followed by addition of 1 mL of virus and 3 mL of medium such that the total volume equaled 4 mL. After 72 h, 78–86% of cells were GFP positive, as detected by flow cytometry. Selection was then performed by puromycin treatment at 0.5 µg/mL for JR1 cells, and 1 µg/mL for Rh41 cells. The media was changed and cells were split twice a week for 10 days, after which cells were used for the below assays.

### 2.3. Exosome Isolation

Exosomes were isolated as previously described [15]. Briefly, exosome-free (exo-free) medium was prepared by ultracentrifugation of 40% FBS/DMEM at 100,000× *g* overnight at 4 °C. The resulting supernatant was filtered with 0.22 μm filter (Millipore, Darmstadt, Germany) and then diluted in a ratio 1/4 to obtain exo-free medium with 10% FBS. JR1 and Rh41 cells were cultured in exo-free medium for 72 h at 37 °C in 5% CO_2_ in 15 cm plates. The culture medium was collected and centrifuged 3 times at increasing speeds (300× *g* for 10 min, 2000× *g* for 20 min, and 10,000× *g* for 30 min). The supernatant was collected in ultracentrifuge tubes, mixed with Exoquick solution (SBI, Mountain View, CA, USA) for exosome precipitation, and stored overnight at 4 °C. The solution was ultracentrifuged at 100,000× *g* for 70 min at 4 °C to remove contaminating elements. The pellet was collected, washed with 700 μL PBS, then centrifuged at 100,000× *g* for 70 min. The final pellet was resuspended in 300μL PBS for exosome use in functional assays, in lysis buffer for protein extraction, or in Trizol^®^ for RNA extraction.

### 2.4. Protein Extraction and Analysis

Proteins were extracted from transduced cells using RIPA 1X lysis buffer (20 mM Tris-HCl pH 7.5; 150 mM NaCl, 1 mM Na2EDTA, 1 mM EGTA, 1% NP-40, 1% sodium deoxycholate, 2.5 mM sodium pyrophosphate, 1 mM β-glycerophosphate, 1 mM Na_3_VO_4_, 1 µg/mL leupeptin). For protein extraction from exosomes, CHAPS lysis buffer (30 mM Tris–Cl, pH 7.5; 150 mM NaCl; and 1% CHAPS) mixed with 25X protease inhibitor (Roche, Basel, Switzerland) was used. The mixture was sonicated for 15 min, centrifuged for 10 min at 13,000× *g* at 4 °C, then the supernatant containing the proteins was collected. Proteins were quantified using a Bradford assay and the absorbance was read at 595 nm on an ELISA plate reader. The concentration of the proteins was determined with respect to known protein standard concentrations of Bovine Serum Albumin (BSA) (Sigma-Aldrich, Dorset, UK). Western blotting was performed using 12% acrylamide gel. Using equal amounts of proteins (25 µg for all exosome lanes, and 40 µg for all cell lysate lanes), loading buffer (Tris-HCl 0.25 M, pH 6.8; SDS 4%; Glycerol 20%; bromophenol blue and 5% β-mercaptoethanol) was added to each sample. Migration was allowed to take place at 90 V for the stacking gel, then at 120 V for the resolving gel. Transfer to nitrocellulose membrane (Santa Cruz Biotechnology, Heidelberg, Germany) was done in TGS1X-10% methanol transfer buffer for 90 min at 350 mA. The membrane was blocked to prevent non-specific binding by using 3% BSA-TBS1X-0.001% Tween (Tris (hydroxymethyl); NaCl; KCl and Tween 20; pH = 7.5). The membrane was incubated with specific primary antibody diluted in 3% BSA-TBS1X-0.001% Tween either for 2 h at room temperature or overnight at 4 °C, then washed 3 times by TBS1X-0.001% Tween for 5 min before adding the corresponding horseradish peroxidase (HRP)-conjugated mouse anti-rabbit secondary antibody (Santa Cruz Biotechnology, Heidelberg, Germany) diluted in 3% BSA-TBS1X-0.001% Tween for 1 h. The membrane was developed using ChemiDoc (Bio-Rad Laboratories, Hercules, CA, USA) after adding Clarity Western ECL reagent (Bio-Rad, Hercules, CA, USA) as a substrate. Bands were quantified using ImageJ^®^ software (Version 1.53e, NIH, Bethesda, MD, USA). The primary antibodies used were: anti-CD147, anti-GAPDH, anti-Calnexin, anti-caspase 3, anti-Bcl2, and anti-VEGF (all from Santa Cruz Biotechnology, Heidelberg, Germany), anti-TSG101 (Abcam, Cambridge, UK), anti-phospho-ERK and anti-ERK (Cell Signaling Technology, Danvers, MA, USA).

### 2.5. Reverse Transcription Real-Time Polymerase Chain Reaction (RTq-PCR)

Total RNA was extracted using Trizol^®^ reagent (Life Technologies, Carlsbad, CA, USA) according to the manufacturer’s instructions and treated with DNase I (Qiagen, Hilden, Germany). cDNA was synthesized using a RevertAid first-strand cDNA synthesis kit (ThermoScientific, Vilnius, Lithuania). Real-time PCR was done with the iQ SYBR green supermix kit in a CFX96 system (Bio-Rad Laboratories, Hercules, CA, USA). Amplification was performed using the following primers: GAPDH sense, AGCCAAAAGGGTCATCATCT; antisense, GGGGCCATCCACAGTCTTCT; CD147 primer pair: sense, GGCTGTGAGTCGTCAGAACAC; antisense, ACCTGCTCTCTCGGAGCCGTTCA. PCR conditions included denaturation at 95 °C for 15 min, 40 cycles of 95 °C for 15 s, 72 °C for 1 min, and then annealing at 55 °C. GAPDH was used as an endogenous control. Experiments were done in triplicate using a CFX96 real-time PCR detection system (Bio-Rad Laboratories, Hercules, CA, USA), and data analysis was performed using the ΔΔCT method.

### 2.6. Cell Viability, Colony Formation, and Scratch Assays

For the MTT cell viability assay, 15,000 cells were seeded onto 96-well plates and cultured as above. The following day, medium was replaced by exo-free medium with exosomes. Control cells were incubated in exo-free medium. MTT cell viability assay (Roche Life Sciences, Penzberg, Germany) was performed according to the manufacturer’s instructions. Results were computed as the mean percent absorbance of exosome-treated condition relative to control.

For colony formation assay, cells transduced with *shCD147* or *shScr* control were plated at a density of 15,000 cells/plate onto 6-well plates coated with 1.5 mL of 0.8% agar. Cells were mixed with 1 mL complete medium containing 0.48% agar and added as a top layer. 1 mL complete medium was added to each well every 2 days to prevent drying, and incubated at 21% O_2_, 5% CO_2_. After 10 days, colonies were photographed and counted. To quantify the size of the colonies, the area of each individual colony (in pixels) was measured by ImageJ^®^ software (Version 1.53m, NIH, Bethesda, MD, USA).

For in vitro scratch assay, cells were seeded in a 24-well plate and incubated at 37 °C until they reached 80–90% confluence. A scratch/wound was created vertically at the center of the well using a 200 μL pipette tip, and the dead cells were washed with phosphate-buffered saline (PBS). Serum-free DMEM was added. Images were taken in randomly selected fields at 0, 8, and 24 h using an inverted light microscope at 10× magnification. To obtain the same field during the image acquisition, markings were used as reference points close to the scratch. For each image, distance between one side of scratch and the other (width) was measured in μm at indicated time using ImageJ^®^ software (Version 1.53m, NIH, Bethesda, MD, USA).

### 2.7. Cell Proliferation, Transwell Migration, and Transwell Invasion Assays

Human BJ fibroblasts were seeded onto a 24-well plate with 50,000 cells per well in 1 mL medium and incubated at 37 °C for 4 h. The medium was aspirated, and exosomes were added in 1 mL exo-free medium and incubated for 72 h. Then, cells were washed with PBS, trypsinized collected, and counted using a hemocytometer. For the transwell migration assay, D Falcon™ Cell Culture Inserts with 8μm pore size were used, and the same inserts coated with 10% growth factor-reduced matrigel were used for the invasion assay (BD Biosciences, Bedford, MA, USA). Human BJ fibroblasts were seeded onto the top chamber (50,000 cells per insert) in 300 μL exo-free medium and 500 μL of serum-free medium was added into the bottom chamber in a 24-well plate. Exosomes were added 4 h later onto the top chamber. Control cells were incubated in exo-free medium. The inserts were fixed after 24 or 72 h, stained with hematoxylin and eosin, mounted, cover slipped, air-dried and photographed, and the migrating/invading cells counted using ImageJ software.

### 2.8. Uptake of CFSE-Labeled RMS-Derived Exosomes by Fibroblasts

Exosomes from each condition were fluorescently labeled by a 20 μM concentration of carboxyfluoresceine diacetate succinimidyl-ester (CFSE) dye by incubating for 30 min at room temperature and in darkness. To stop labelling, approximately 5-fold volume of cell culture medium was added to the solution and ultracentrifuged at 100,000× *g* for 70 min at 4 °C to eliminate the unincorporated dye. The pellet was resuspended in filtered PBS, and exosomes were quantified as protein concentration by means of a Bradford assay. BJ fibroblast cells were seeded at a density of 50,000 cells/well in a 24-well plate 24 h prior to exosome addition with normal growth medium at 37 °C in a 5% CO_2_ incubator. The next day, cells were treated with equal amounts (2 μg) of CFSE-labeled exosomes of each condition, in exo-free medium, incubated for 24 h, washed twice with PBS, then stained with Hoechst dye (20 μM). Pictures were taken on a Microscope Zeiss Axio (Carl-Zeiss, Dresden, Germany) at 20× magnification. Fluorescence intensity was measured by ImageJ^®^ software, using the pixel count tool, to quantify the amount of exosomes taken up by adherent cells.

### 2.9. Immunohistochemical Staining of Human Tumor Samples

All human studies were approved by the Institutional Review Board (IRB) at the American University of Beirut Medical Center (AUBMC). A total of 46 archived formalin-fixed paraffin-embedded rhabdomyosarcoma tumor samples were identified for patients younger than 30 years of age, collected at the AUBMC over the period 2002–2018. Of those, 28 samples had enough material for sectioning and immunostaining, and were therefore included in the study. In addition, 5 samples were identified for patients younger than 30 years of age diagnosed between the period 2016–2020 at the Institut National de Pathologie in Beirut, Lebanon, bringing the total to 33 eligible samples. Clinical characteristics were linked to the tumor samples, with 26 being localized and 7 metastatic. Paraffin-embedded tumors were sectioned at 4 µm. Antigen retrieval was performed in a steamer using citrate antigen retrieval buffer (pH 6.0). Staining was performed using anti-CD147 (Santa Cruz Biotechnology, Heidelberg, Germany), using an ABC Elite Kit (Vector Labs, Burlingame, CA, USA) according to the manufacturer’s protocol for detection by biotinylated secondary antibody and streptavidin conjugated to horseradish peroxidase followed by DAB substrate (DAKO, Glostrup, Denmark). Tissues were viewed under a light microscope at 40× magnification. Intensity of CD147 expression was scored as negative (0), weak (1), moderate (2), and high (3), by an experienced pathologist, while blinded to patient clinical characteristics or metastatic stage. Positive tumors were then given a second score based on percent of positive cells, with tumors that had >75% of cells positive receiving a score of 4, 50–75% a score of 3, 25–50% a score of 2, and <25% a score of 1. The product of the first and second scores constituted the CD147 positivity score.

### 2.10. Statistical Analysis and Imaging

All in vitro experiments were performed in biological and technical triplicate unless mentioned otherwise. Comparisons between experimental groups were performed using Mann–Whitney U test, except for categorical variables (positive versus negative staining of human tumor samples), where Fisher’s exact test was used. For all tests, a *p*-value of less than or equal to 0.05 was considered statistically significant. A box plot was created using Microsoft Excel ® (version 2019) where each box represents the interquartile range (25th to 75th percentiles), the central horizontal line represents the median value, and the whiskers represent the range. All data is presented as mean ± standard deviation. Digital photomicrographs were obtained using a Zeiss Axio Observer Z1 microscope. Composite images were constructed using Adobe Photoshop CS6 ® software (Version 13.0 ×64, Adobe Systems, San Jose, CA, USA).

## 3. Results

### 3.1. CD147 Is Expressed in RMS Cells, and Its Knockdown Diminishes RMS Cell Invasive Properties

**Figure 1:**
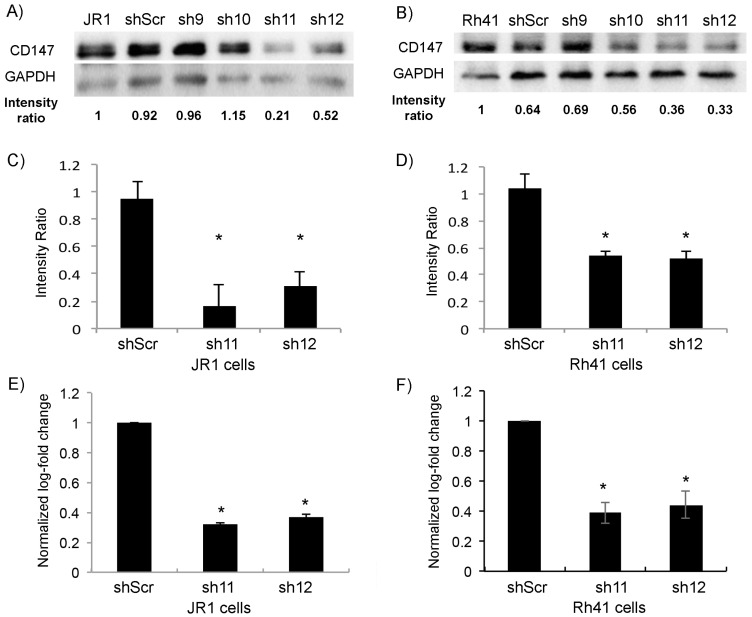
Transduction of JR1 and Rh41 cells with lentiviral vector expressing shRNA-CD147 downregulates its expression in cells. (**A**,**B**) Western blot for the indicated proteins in JR1 (**A**) and Rh41 (**B**) cells transduced with four different shRNAs targeting *CD147*; *shScr* is used as a control. Band intensity ratios relative to loading control GAPDH are shown, normalized to the control conditions. (**C**,**D**) Quantitation of the band intensity ratios as in (**A**,**B**), with means computed across three western blot experiments. (**E**,**F**) Bar charts representing *CD147* RNA levels normalized to *GAPDH* in both JR1 (**E**) and Rh41 (**F**) cells. Bars represent standard deviation between triplicates. Asterisks (*) denote a statistically significant difference (*p*-value ≤ 0.05).

Using the RMS cell lines JR1 and Rh41, derived from an ERMS and an ARMS tumor, respectively [35,36], and using four different shRNA targeting CD147 (*shCD147*), and scrambled construct (*shScr*) as negative control, we found that the two constructs *shRNA-11* (*sh11*) and *shRNA-12* (*sh12*) exhibited the highest knockdown of CD147 by both protein expression (Figure 1A,B, with quantitation in Figure 1C,D), and RTq-PCR (Figure 1E,F), and therefore these two shRNA constructs were subsequently used throughout the remainder of this study.

**Figure 2:**
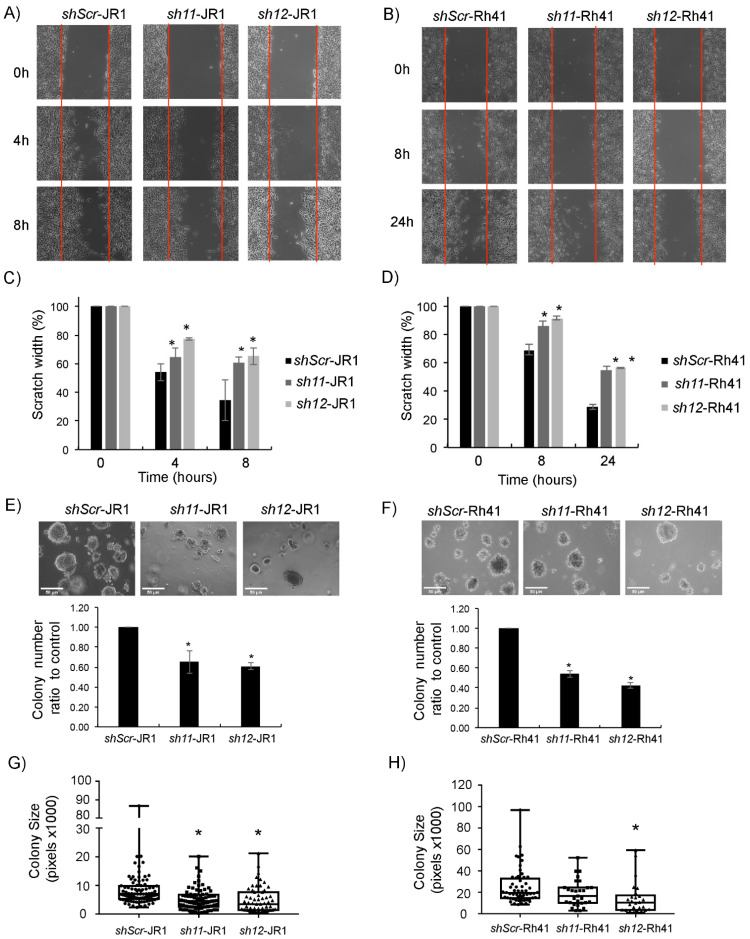
*CD147* knockdown affects RMS cell phenotype. (**A**,**B**) Representative images of scratch assay performed on JR1 (**A**) and Rh41 (**B**) transduced cells. (**C**,**D**) Bar charts represent scratch width (%) of RMS-sh11 and sh12 relative to the control (sh-Scr). (**E**,**F**) Representative images of JR1 (**E**) and Rh41 (**F**) transduced colonies in soft agar. Bar charts represent the number of colonies of RMS-sh11 and sh12 relative to the control. Bars represent standard deviation between triplicates. (**G**,**H**) Representative box plot analysis of the range of sizes of colonies in the different depicted conditions. Asterisks (*) denote a statistically significant difference (*p*-value ≤ 0.05).

To investigate the effect of CD147 knockdown on RMS cell migration, we performed an in vitro scratch assay [37]. Knockdown of *CD147* in JR1 and Rh41 cells significantly decreased migration compared with cells transduced with shambled construct (Figure 2A–D). To investigate effects on invasion, we performed anchorage-independent growth assays by culturing transduced cells in soft agar. After one week, the colony numbers of *CD147* knockdown cells were significantly lower than the numbers of control transduced cells (Figure 2E–F). In addition to a lower number of colonies, the size of the individual colonies was also significantly smaller in JR1 cells (Figure 2G), while for Rh41 cells the size difference tended to be smaller, reaching statistical significance with one of the shRNA constructs (Figure 2H).

**Figure 3:**
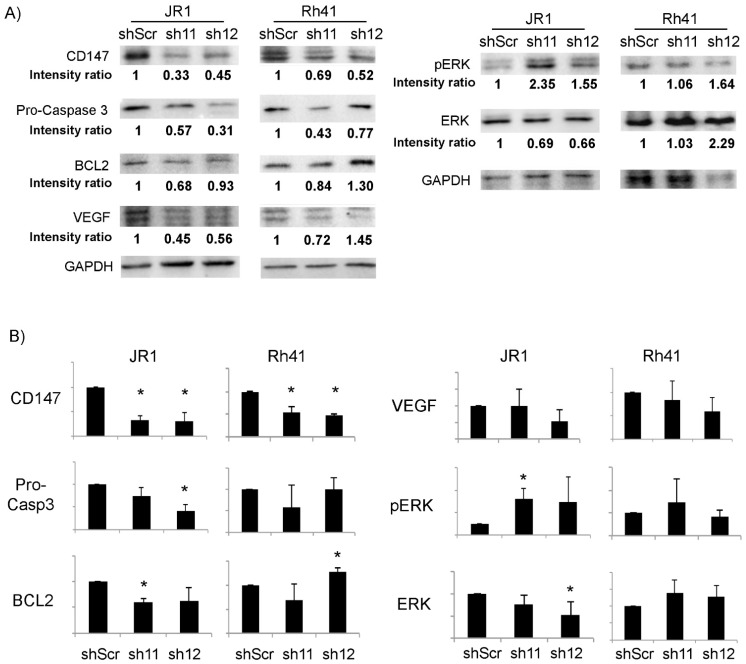
(**A**) Western blot for the indicated proteins in JR1 and Rh41 cells transduced with sh-11 and 12. Intensity ratios relative to loading control GAPDH, normalized to control condition. (**B**) Quantitation of the band intensity ratios as in (**A**), with means computed across three Western blot experiments. Bars represent standard deviation between triplicates. Asterisks (*) denote a statistically significant difference (*p*-value ≤ 0.05).

To determine the effect of CD147 downregulation on growth and survival pathways in RMS cells, we evaluated the expression levels of proteins that have been previously demonstrated to be impacted by CD147 in other cell types [38,39]. As shown in Figure 3, there was a significant reduction in pro-Caspase 3 and BCL2 upon downregulation of *CD147* by one of the two utilized shRNA in JR1 cells, but only a downward trend by the second shRNA, precluding a definitive conclusion but suggesting an effect on anti-apoptotic pathways, while there was no discernible effect in Rh41 cells. Similarly, ERK phosphorylation tended to increase in response to *CD147* knockdown in JR1 cells, but reached statistical significance for only one of the two utilized shRNA constructs, while there was no effect seen in Rh41 cells. VEGF levels were not reproducibly altered in either cell line (Figure 3B). Thus, the interrogated pathways do not seem to explain the effect of CD147 on enhancing RMS cell proliferation, migration or invasion.

### 3.2. Knockdown of CD147 Modulates the Effect of RMS-Derived Exosomes on Recipient Fibroblasts

**Figure 4:**
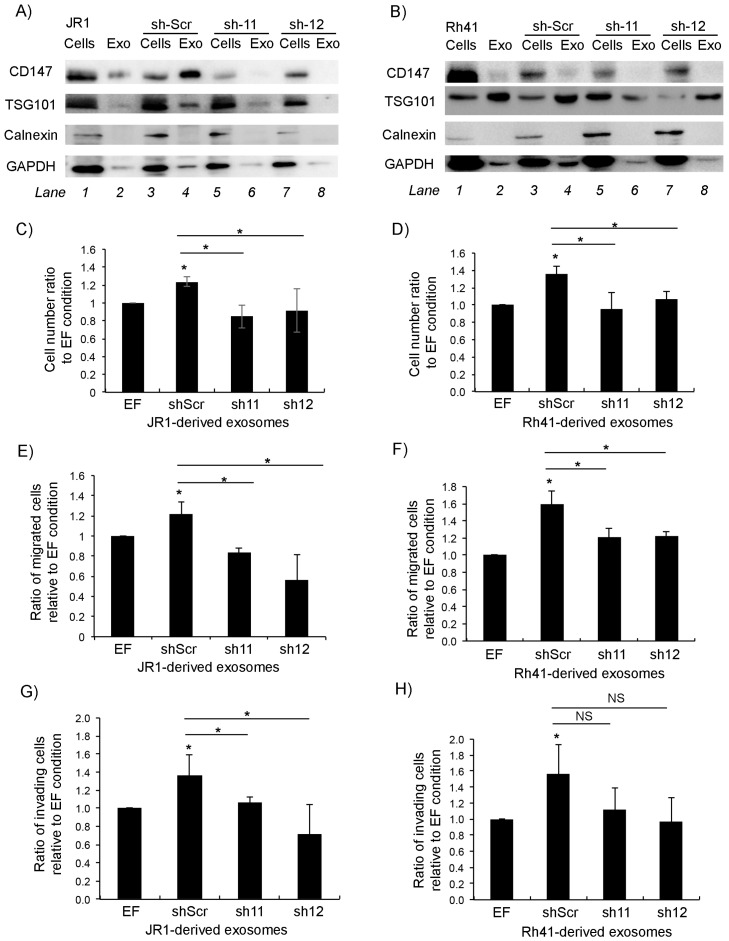
*CD147* downregulation reduces exosome-mediated BJ fibroblast proliferation, migration and invasion. (**A**,**B**) Western blot for the indicated proteins in cells and exosomes derived from JR1 (**A**) and Rh41 (**B**) cells transduced with *shRNA-11* or *-12*; *shRNA-scr* was used as negative control. (**C**,**D**) Bar charts representing the ratio of viable BJ fibroblasts after treatment with exosomes isolated from the indicated transduced JR1 (**C**) or Rh41 (**D**) cells relative to the control (EF). (**E**,**F**) Bar charts showing the ratio of BJ-migrated cells after treatment with exosomes isolated from transduced JR1 (**E**) or Rh41 (**F**) cells relative to the EF control condition. (**G**,**H**) Bar charts representing the ratio of invading BJ fibroblasts after treatment with exosomes isolated from transduced JR1 (**G**) or Rh41 (**H**) cells relative to the EF control condition. Bars represent standard deviation among triplicates. Asterisks (*) denote a statistically significant difference (*p*-value ≤ 0.05). NS denotes non-significant difference (*p*-value > 0.05).

Since CD147 is secreted in RMS-derived exosomes, we sought to examine the effect of *CD147* downregulation on paracrine signaling of RMS cells. Examination of the protein content in exosomes derived from *sh11*- and *sh12*-transduced JR1 and Rh41 cells showed that CD147 within exosomes decreased upon its suppression in the parent cells (Figure 4A,B).

As expected, treatment of normal human BJ fibroblasts with either the control (scrambled *shRNA* transduced) JR1- or Rh41-derived exosomes resulted in a significant increase in fibroblast proliferation compared to exosome-free control-treated cells (Figure 4C,D). This increase was abolished upon treatment with exosomes derived instead from cells with *CD147* knockdown (Figure 4C,D). Similarly, while there was a significant increase in fibroblast migration when cells were treated with either JR1- or Rh41-derived control exosomes (Figure 4E,F), this was again abolished when fibroblasts were treated with exosomes derived from *CD147* knockdown cells (Figure 4E,F). The same effects were seen when investigating fibroblast invasion through matrigel when treated with JR1-derived exosomes (Figure 4G), whereas for cells treated with Rh41-derived exosomes, the number of invasive cells tended to be lower but did not reach statistical significance (Figure 4H).

**Figure 5:**
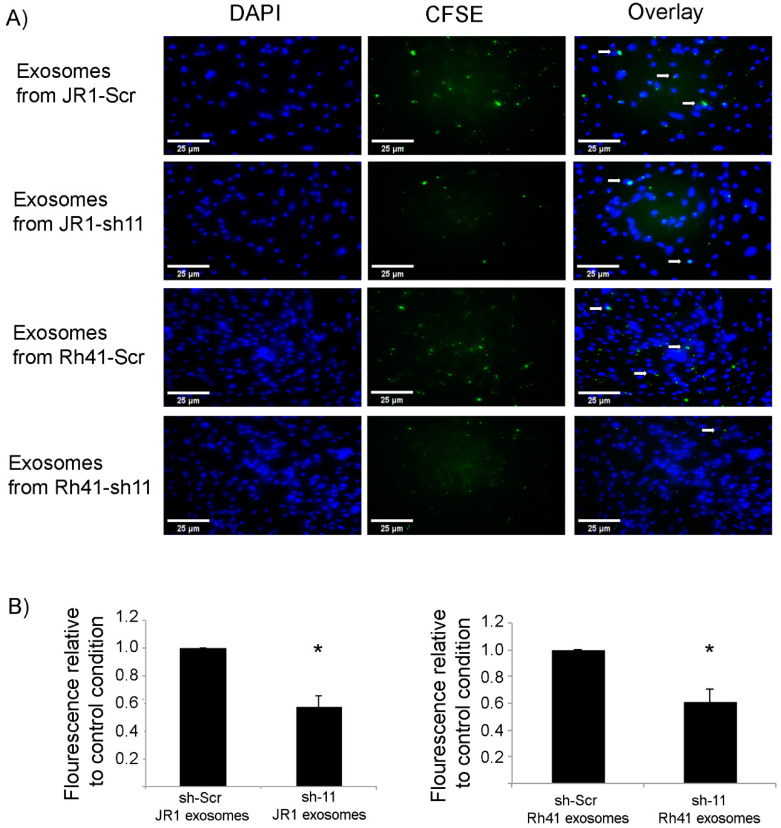
Uptake of fluorescently labeled RMS-derived exosomes by BJ fibroblasts. (**A**) Immunofluorescence microscope images showing the uptake of CFSE-labeled JR1- and Rh41-derived exosomes by BJ fibroblasts treated by the indicated exosomes. White arrows show examples of the green fluorescent exosomes taken up by the adherent cells. (**B**) Bar charts representing the relative fluorescence normalized to the control treatment (exosomes from RMS cells transduced with scrambled shRNA). All settings of image processing were kept constant, and fluorescence intensities were calculated using ImageJ^®^ software. Bars represent standard deviation among triplicates. Asterisks (*) denote a statistically significant difference (*p*-value ≤ 0.05).

We considered whether *CD147* knockdown affected the efficiency of exosome uptake by recipient fibroblasts, as one mechanism for a decreased paracrine effect could be through decreased uptake of exosomes. Indeed, when staining exosomes with the CFSE fluorescence marker and treating recipient fibroblasts with equal amounts of exosomes, we found that uptake of exosomes was decreased by around 50% when they were derived from *CD147* knockdown RMS cells (Figure 5A,B).

### 3.3. Expression of CD147 in RMS Tumors Correlates with the Metastatic Stage

**Figure 6:**
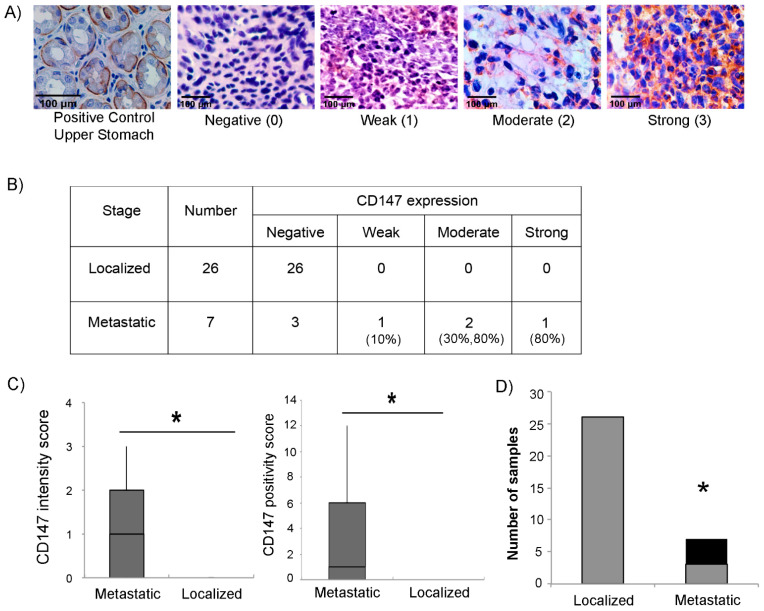
CD147 expression in RMS human tumors. (**A**) Representative images of RMS tissues showing the different intensities used to score CD147; negative (0), weak (1), moderate (2), and strong (3) at 40× magnification. Upper stomach tissue was used as a positive control. (**B**) Table showing the intensity of CD147 expression according to the different stages of RMS clinical tumors. Numbers in parenthesis denote the approximate percentage of total cells staining positive in each respective sample. (**C**) Left panel: box plot representing the range of IHC CD147 intensity score between metastatic and localized RMS tissues (total of 33 tissues); right panel: box plot representing the range of IHC CD147 positivity score between metastatic and localized RMS tissues (total of 33 tissues). (**D**) Number of tumors staining either negative (gray columns) or positive (black columns), by localized versus metastatic stage. Asterisks (*) denote a statistically significant difference (*p*-value ≤ 0.05).

To explore whether CD147 expression correlates with the aggressiveness of RMS tumors, we analyzed the expression of CD147 by immunohistochemistry in 33 human RMS tumor tissues (26 non-metastatic and 7 metastatic). Intensity of CD147 expression was scored as negative (0), weak (1), moderate (2), or high (3), as shown in Figure 6A. Results are shown in Figure 6B, stratified by metastatic group. Interestingly, while four of the seven metastatic tumors scored positive, none of the 26 localized tumors expressed CD147. Notably, immunoreactivity against CD147 was observed predominantly in the cytoplasm and/or stroma of tumor tissue with some membranous staining, unlike the typically observed membranous stain in normal cells (Figure 6A). CD147 positivity showed a statistically significant association with metastatic stage, whether by intensity score (Figure 6C, left panel), positivity score (computed as a composite of intensity and extent of positive cells within the tissue) (Figure 6C, right panel), or by simple designation as positive or negative stain (Figure 6D).

## 4. Discussion

Aggressive disease and distant metastasis are current challenges that hinder treatment strategies in rhabdomyosarcoma patients where overall survival outcomes remain unsatisfactory [40]. This highlights the need for identifying novel therapeutic targets for effective treatment, especially of locally invasive and metastatic disease.

CD147, a member of the immunoglobulin superfamily [27], is highly expressed on the surface of various types of cancer cells, including esophageal cancer, where it has been associated with worse survival outcomes and poor prognosis [41]; bladder cancer, where it correlates with cancer cell proliferation [42]; and breast cancer, where CD147 expression is an indication of increased invasive capacities, presence of metastasis and disease recurrence [43]. We identified CD147 to be expressed in RMS-derived exosomes across a panel of cell lines, which suggested that it may have a role in RMS paracrine signaling, potentially contributing to the known invasiveness and metastatic propensity of this tumor. Exosomes rich in CD147 have been identified in epithelial cancers such as ovarian and colorectal cancer, and found to be associated with higher stages of disease [34], and may promote angiogenesis in endothelial cells [44].

Our work shows that CD147 expression in RMS cancer cells contributes to cellular invasive and migration abilities, and its knockdown was also able to reduce the formation of clones in vitro, suggesting a role in promoting cancer cell stemness. This is consistent with the effect of CD147 knockdown in epithelial cancer types, where it was shown to reduce migration and clonogenic growth [45,46].

In our investigation, we could not demonstrate a reproducibly significant decrease in Bcl2 and pro-caspase-3 upon CD147 suppression, nor a reproducibly significant change in ERK phosphorylation, though the trends correlated with prior reports. Kulyar et al. had previously shown that chondrocytes exhibit enhanced survival when CD147 expression is elevated, which was in part associated with an upregulation of Bcl2 and downregulation of caspase-3 in their system [47]. In hepatoma cells, CD147 was also shown to protect cells from apoptosis by upregulating Bcl2 levels and promoting ERK signaling [48]. Also, our work showed that in RMS cell lines, there was no significant change in VEGF expression upon CD147 downregulation, unlike results observed in other cell lines, such as lung adenocarcinoma where CD147 can upregulate VEGF at both the mRNA and protein levels [28]. CD147 inhibition has also been associated with a decrease in VEGF in breast cancer [49], melanoma [50] and acute myeloid leukemia [51]. Further experiments are therefore needed to identify the specific downstream effectors of CD147 in RMS cells, preferably through an unbiased evaluation of downstream signaling pathways through RNA-sequencing and proteomic profiling.

The crosstalk between tumor and neighboring cells, such as fibroblasts and endothelial cells, plays a crucial role in the development and progression of tumors [52,53]. In this regard, CD147 expressed on cancer cells has been proven to stimulate the adjacent stromal cells to produce several MMPs altering the stromal microenvironment by modifying extracellular matrix composition which aids in tumor growth and invasion. CD147 is released from cancer cells into the tumor microenvironment either in a soluble form or associated to extracellular vesicles [54,55]. Previous studies have shown that shRNA-mediated knockdown of *CD147* in malignant melanoma cells, followed by treatment of fibroblasts with the corresponding microvesicles and exosomes, decreased MMPs’ enzymatic activity in recipient fibroblasts, suggesting that extravesicular CD147 downregulation is associated with decreased extracellular matrix remodeling and metastasis [56]. In our study, we provide evidence that exosomes shed by RMS cells stimulated invasive properties of normal human fibroblasts, and that this is at least partially mediated by CD147 and its downstream signaling. As such, CD147 may promote tumor invasiveness by acting either directly or indirectly on neighboring stromal cells, promoting pathways that enhance cell growth and motility. At least some of these effects are mediated through improving the efficiency of RMS-derived exosome uptake by recipient cells, though the exact mechanism by which exosome uptake is enhanced remains unclear. This is especially relevant given that we found that CD147 was exclusively expressed (by immunohistochemical staining) in primary RMS tumor samples of patients with metastatic disease. Interestingly, CD147 staining was observed mainly in the tumor stroma, which supports its role in the tumor microenvironment and the tumor/stroma cells’ crosstalk. In other types of cancer CD147 is also correlated with disease stage, such as in gastric cancer, where its expression is elevated compared to adjacent normal tissues and is associated with the metastasis and TNM stages [57]. In pulmonary adenocarcinoma, CD147 expression also correlates with lymph node metastasis and can act as a prognostic biomarker of the advanced stage [58].

In our study, we used two representative RMS cell lines, one of ERMS (and fusion gene negative) and one of ARMS (and fusion gene positive) histology. The reproducibility of the findings across the two histologically distinct RMS cell lines, as well as the compelling data from the primary human tumor samples, suggest that CD147 is indeed a modulator of invasive properties in RMS. Based on our findings in the primary human tumor samples, and since the RMS cell lines were initially derived from metastatic tumors [36], we expect that CD147 expression is likely to be relevant primarily to metastatic tumors. However, verification will require evaluation of CD147 expression and the effect of its down- or upregulation, in a panel of primary cultures from metastatic versus localized RMS tumors, availability of which is currently limited by the relative rarity of the disease [59].

In conclusion, we have shown that CD147 enhances RMS tumor cell survival and growth and that RMS-derived exosomes harbor CD147 and stimulate fibroblast cell proliferation and invasion, which supports the hypothesis that paracrine signaling modulated by CD147 plays a role in tumor progression and metastasis. Further studies will focus on identifying the specific downstream effects of CD147 in both RMS cells as well as the RMS tumor microenvironment, for its potential investigation as a therapeutic target for aggressive and/or metastatic disease.

## Data Availability

The data presented in this study are available within the article and upon request.
